# Health Tract, No. 82

**Published:** 1862-07

**Authors:** 


### HEALTH TRACT, No. 82.

### MILK—ITS USES.

MILK is the natural and all-sufficient food of infancy, containing as it does all the elements of nutrition necessary for sustaining, repairing, and building up the new being; but as the little one gets the power to move its muscles, then crawl, and walk, and run, so much of the more solid portions of the body are worked out or used up by the friction of the complicated machinery, that milk alone can not supply the rapid wastes, and the instincts of the child call out in very decisive tones for more substantial aliment, and it greedily eats bread and meat. Nature herself weans the child from the all-absorbing love of milk, showing that it is the natural food only of infancy. The active and laborious, whether as to body or brain, soon find that they must have something more "solid" than milk.

Except in rare cases, milk as a chief or even large article of diet, is most pernicious to the sickly, the infirm, and the convalescent. And under any and all circumstances, it is necessary, when all its healthful and natural qualities are desired to be secured, as an aliment, that it should be drank while warm from its natural fountain; because, as soon as it loses its natural heat, it dies, it begins to decay, to decompose, unless, when milked, it is stirred well, until cooled, and then is put in a very cold place, or enveloped with ice, so that it shall neither freeze nor be warmer than fifty degrees. M. Flourens, a distinguished French physiologist, found in 1861, that if the animal mother is fed with madder, her own bones become tinged with its color, and also at length those of sucklings, although having no connection with the mother, except while at the breast. It would seem then that the body, the constitution of the suckling, is affected by what the parent eats and drinks. It therefore follows that the cow or other animal whose milk we use, should be healthy and should live on healthful food and in a *natural manner!* But a cow confined on ship-board, in the stable of a private citizen, or in the narrow stenchy stalls of the milkeries which supply cities, does not live a natural life, and can not by any possibility give natural, healthful milk; hence chemists and microscopists assure us, that when the milk of a confined cow is minutely examined, even immediately after milking, it exhibits globules of yellow matter, such as come from sores and ulcers. If this is true, it is a disgusting thought, and would seem to prove that no family ought to use milk, unless drawn from cows which roam in luxuriant pastures from one day's end to another, and that breathe a pure atmosphere winter and summer.

The infant feeds at the breast of its one mother; it would seem natural that when cow's milk is substituted, it should be from the same cow. It is reasonable to suppose, then, that bad milk is an agency of disease and death to multitudes, and especially of children in large cities; particularly in summer-time. Daniel E. Delavan, Esq., City Inspector of New-York, in his admirably arranged annual report shows, that of twenty-two thousand persons who died during 1861, three thousand three hundred were children under two years of age! Six thousand affectionate hearts lacerated beyond healing, for all time! It can not be known definitely what proportion of all this death and sorrow is traceable to bad milk, but that it is an important item can not be well disputed. Whatever it is, is avoidable simply by encouraging those milk-furnishing companies who, 1st, Deliver milk from cows fed in field and pasture. 2d. Who deliver milk daily to any one desiring it, from the same cow. 3d. Who cool the milk at the time it is drawn, and keep it cool until it is delivered at our doors for consumption. One company at least does this, *the Rockland County and New-Jersey Milk Association*, at 146 Tenth street, near Broadway, New-York, under the vigilant management of S. W. Canfield, Esq.

